# MEASURING LIGHTING USING AN INDIVIDUAL WEARABLE LIGHT SENSOR IN NURSING HOME RESIDENTS WITH DEMENTIA

**DOI:** 10.1093/geroni/igae098.2591

**Published:** 2024-12-31

**Authors:** Ying-Ling Jao, Julian Wang, Shevvaa Beiglary, Yo-Jen Liao

**Affiliations:** The Pennsylvania State University, University Park, Pennsylvania, United States; The Pennsylvania State University, University Park, Pennsylvania, United States; The Pennsylvania State University, University Park, Pennsylvania, United States; The Pennsylvania State University, University Park, Pennsylvania, United States

## Abstract

More research has examined the light effect in dementia. Lighting exposure varies widely across times and people. Individual light sensors allow more precise measurements. Yet, their feasibility has not been established. This study examined the feasibility of using individual light sensors (LYS Button) in a clinical trial for nursing home residents with dementia. This study enrolled 27 residents with dementia. The LYS Button is a small, lightweight sensor clipped on residents’ collar/shoulder area. The sensor measured lighting every 15 seconds all day, 7 days a week, every other week for 13 weeks. Several strategies were implemented. First, instructions on sensor use were provided, and a reminder sign was posted in the residents’ bedrooms. Second, a computer station on site enabled data monitoring. If a sensor disconnected from the server, the team was alerted, and on-site staff were contacted to check/replace sensors. Third, the research team checked the sensor on-site twice weekly. To examine the feasibility, the number of sensors was tallied, sensor data were analyzed, and individual interviews were conducted with 22 healthcare workers. Results showed that 52 sensors were lost or broken. The average missing data per resident per day were 1.13 (SD=0.99) and 6.84 hours (SD=2.28) during daytime and nighttime, respectively. Despite some missing data, the intense data collection allowed sufficient data for analysis. The results support the feasibility of using the wearable sensor to measure daytime lighting. Wearing methods and strategies during nighttime need modification to improve the use of individual lighting sensors in people with dementia.

